# Long-term effects of vitamin D supplementation in vitamin D deficient obese children participating in an integrated weight-loss programme (a double-blind placebo-controlled study) – rationale for the study design

**DOI:** 10.1186/s12887-017-0851-7

**Published:** 2017-04-04

**Authors:** Agnieszka Szlagatys-Sidorkiewicz, Michał Brzeziński, Agnieszka Jankowska, Paulina Metelska, Magdalena Słomińska-Frączek, Piotr Socha

**Affiliations:** 1 Department of Paediatrics, Gastroenterology, Hepatology and Nutrition, Medical University of Gdańsk, ul. Nowe Ogrody, 1-6 80-803 Gdańsk, Poland; 2 Department of Public Health and Social Medicine, Medical University of Gdansk, Zwycięstwa 42a, 80-210 Gdansk, Poland; 3 “6-10-14 for Health” University Clinical Centre, Dębinki 7, 80-210 Gdansk, Poland; 4 Pomeranian Medical Centre, Nowe Ogrody 1-6, 80-803 Gdansk, Poland; 5 Department of Gastroenterology, Hepatology and Feeding Disorders, The Children’s Memorial Health Institute, Dzieci Polskich 20, 00-999 Warsaw, Poland

**Keywords:** Vitamin D, Obesity, Weight loss, Body composition

## Abstract

**Background:**

Obesity is associated not only with an array of metabolic disorders (e.g. insulin resistance, hiperinsulinemia, impaired tolerance of glucose, lipid disorders) but also skeletal and joint abnormalities. Recently, a pleiotropic role of vitamin D has been emphasized. Obese children frequently present with vitamin D deficiency, and greater fat mass is associated with lower serum concentration of this vitamin. Although some evidence suggests that weight loss may affect vitamin D status, this issue has not been studied extensively thus far. The aim of a double-blind placebo-controlled study is to assess long-term health effects of vitamin D supplementation in vitamin D deficient obese children participating in an integrated weight-loss programme.

**Methods:**

A randomized double-blind, placebo-controlled trial analysing the effects of vitamin D3 supplementation in overweight or obese vitamin D deficient (<30 ng/ml) children participating in an integrated weight-loss programme. Children are randomized to receive either vitamin D (1200 IU) or placebo for 26 weeks. Primary endpoints include changes in BMI (body mass index), body composition and bone mineral density at the end of the study period, and secondary endpoints – the changes in laboratory parameter reflecting liver and kidney function (transaminases, creatinine) and glucose homeostasis (glucose and insulin levels during oral glucose tolerance test).

**Discussion:**

The effects of vitamin D supplementation in obese individuals, especially children, subjected to a weight-loss program are still poorly understood. Considering physiological processes associated with puberty and adolescent growth, we speculate that supplementation may enhance weight reduction and prevent bone loss in obese children deficient in this vitamin.

**Trial registration:**

NCT 02828228; Trial registration date: 8 Jun 2016; Registered in: ClinicalTrials.gov. The trial was registered retrospectively.

## Background

Obesity is associated with an array of metabolic disorders, such as insulin resistance, hiperinsulinemia, impaired glucose tolerance, abnormal fasting glycaemia, symptomatic diabetes mellitus, dyslipidaemia and cardiovascular disorders, namely arterial hypertension. Moreover, overweight or obese subjects are at increased risk of joint and skeletal disorders, respiratory problems, kidney diseases and alimentary dysfunction, especially non-alcoholic fatty liver disease. High risk of complications associated with childhood obesity justifies early implementation of intervention programmes. Many previous studies demonstrated that the most effective form of intervention are integrated multidisciplinary weight-loss programmes, involving not only children but also their family members [1–3]. Reduction of fat mass is associated with normalization of metabolic parameters, such as inflammatory markers, lipid profile, insulin resistance and arterial blood pressure [3–6]. Thus, early effective intervention increases the likelihood of staying healthy at older age.

Role of vitamin D in energetic metabolism has been emphasized quite recently. Many obese children present with low blood concentrations of vitamin D [7–9], probably due to its insufficient dietary intake and too small amount of outdoor physical activity [10, 11]. Also greater fat mass seems to be associated with lower blood concentration of vitamin D, which may, at least partially, result from sequestration of this vitamin in adipose tissue [12]. Animal experiments with labelled vitamin D demonstrated that it is accumulated in adipose tissue and slowly released to circulation [13].

Another important issue is contribution of vitamin D to the etiopathogenesis of metabolic syndrome. Previous studies documented inverse relationships between blood concentration of vitamin D, waist circumference, systolic blood pressure, insulin resistance, fasting glycaemia, total cholesterol, triglyceride and LDL cholesterol levels in paediatric subjects, as well as positive associations between concentrations of vitamin D and HDL cholesterol [7, 14, 15]. Vitamin D seems to interfere with insulin secretion both directly, binding to its receptors (VDR) on pancreatic β cells, and indirectly, modulating extracellular concentration of calcium [16].

Importantly, a positive association has been found between concentration of vitamin D and sensitivity to insulin in obese children; furthermore, the level of this vitamin correlated inversely with concentration of glycated haemoglobin (HbA1c) [17]. Moreover, obese children with low concentrations of vitamin D presented with elevated levels of inflammatory mediators, such as cathepsin S, chemerin and soluble vascular cell adhesion molecule (sVCAM); this may imply indirectly that vitamin D acts as an immunomodulator [14]. Although supplementation of vitamin D contributed to a decrease in insulin resistance in obese adolescents, their levels of inflammatory markers (CRP, TNF-α, IL-6) remained unchanged [18].

These findings imply that vitamin D deficient obese children may be at increased risk of many metabolic disorders, such as insulin resistance, hiperinsulinemia, impaired glucose tolerance, abnormal fasting glycaemia, symptomatic diabetes mellitus, dyslipidaemia and arterial hypertension. Indeed, a number of observational studies documented a substantial role of vitamin D deficiency in etiopathogenesis of metabolic syndrome and other obesity-related complications. However, we still lack evidence from interventional studies confirming causal character of these relationships.

Metabolic effects of obesity on bone growth and maturation are still not fully understood. Moreover, the results of previous studies analysing bone mass and bone density in obese individuals are highly inconclusive. While according to some authors, bone mass decreases with body weight [19], others did not document a significant effect of body weight on bone mineral density [20, 21] or even demonstrated that obese children, adolescents and adults presented with relatively higher bone mass and dimensions [22, 23]. An increase in bone mass and bone density in obese subjects is postulated to result from greater mechanical load, direct effect of leptin or enhanced enzymatic activity of aromatase [23–26]. Nevertheless, obesity was also shown to be associated with a marked increase in bone fracture risk in paediatric population [23].

Vitamin D plays an important biological role in the process of bone maturation and mineralization. Previous studies documented an inverse relationship between blood concentration of vitamin D and bone mineral density [27, 28]. In a recently published meta-analysis, supplementation of vitamin D was shown to improve both bone mineral density and total bone mass in subjects deficient with this vitamin [29]. The effects of the supplementation seem to be particularly favourable in premenarcheal girls with normal body weight, in whom administration of vitamin D was shown to result in an increase in both bone mass and fat-free mass [30].

Surprisingly, the results of previous studies analysing a relationship between obesity, vitamin D and bone metabolism are sparse and highly inconclusive. An analysis of 58 morbidly obese teenagers demonstrated that individuals with physiological blood concentrations of parathyroid hormone (PTH) presented with normal bone mineral density, irrespectively of their vitamin D levels [31].

In contrast, a recently published study including a small group of adolescents with obesity (*n* = 24) or normal body weight (*n* = 25) showed that the former presented with higher bone mineral density than their normal-weight peers; this relationship turned out to be independent of blood concentration of vitamin D, physical activity level and fat-free mass content. However, in the same study, bone mineral density correlated with blood concentrations of leptin and insulin [25].

The abovementioned data suggest that further research on the role of vitamin D in bone metabolism of obese individuals may be of vital importance.

Aside from many unquestioned favourable health effects, weight loss may also contribute to enhanced bone turnover and cause a decrease in bone mineral density. The results of a recently published systemic review imply that a decrease in bone mass may be a consequence of a calorie-restricting diet, rather than a result of an exercise-induced weight-loss [32]. However, this evidence originates mostly from studies conducted among adults [33–35] and to the best of our knowledge, the issue in question was a subject of only one study including adolescents after bariatric surgeries [36]. Furthermore, the results of intervention studies suggest that a low-calorie albeit high-protein (ca. 30%) diet, with high amounts of dairy products, may prevent bone mass loss and protect against a decrease in bone mineralization [37].

Available data on the efficacy of vitamin D supplementation in adults subjected to a weight-loss intervention are inconclusive and limited. Although in one study, administration of vitamin D turned out to be associated with more evident decrease in fat mass content in persons being on a slimming diet [38], in another experiment, similar intervention did not exert any effect on total body weight [39, 40]. However, the abovementioned findings are not necessarily contradictory, as the loss of fat mass is not an equivalent of a decrease in total body weight.

Most of the previous studies dealing with the problem in question were observational and centred around an association between vitamin D status and bone mineral density.

Owing a paucity of data on the biological role of vitamin D supplementation during weight-loss intervention in children, we decided to verify this association during a double-blind placebo-controlled randomized trial. The aim of the currently ongoing study is to verify if supplementation of vitamin D to obese children deficient with this vitamin exerts an effect on an outcome of an integrated weight-loss intervention.

## Design

### Study design overview

This is a double-blind placebo-controlled randomized study to asses long-term biological effects of vitamin D supplementation to obese children deficient with this vitamin during an integrated weight-loss intervention. Vitamin D-deficient subjects participating in the intervention have been randomized to two arms, receiving vitamin D (1200 IU) or placebo for 26 weeks.

We hypothesized that supplementation of vitamin D in obese children presenting with low serum levels of vitamin D (25(OH)D3) may exert a beneficial effect on the outcomes of an integrated weight-loss intervention, namely on changes in BMI, muscle mass, bone mass, bone mineral density and biochemical markers of metabolic complications related to obesity.

### Research plan

#### Integrated weight-loss intervention – “6–10-14 for health”

The study is conducted as a continuation and extension of the “6–10-14 for Health” integrated weight-loss intervention programme for obese children from Gdansk municipality. Both participants of the programme and their family members are offered a 12-month integrated intervention, including medical, dietetic and psychological counselling, as well as educational workshops for parents. The same intervention will be offered to all subjects of our study as well.

The first phase of the programme, financed by the municipality of Gdansk, has been conducted in 2011–2013. The programme comprised of a screening and survey conducted among all 6-, 10- and 14-year-old children attending primary and grammar schools. A total of 18,162 children (including 7448 6-year olds, 6720 10-year olds and 3994 14-year olds) were examined. The aim of the screening was to identify a group of children with risk factors of civilization-related disorders, i.e. overweight or obesity defined according to BMI percentile charts developed within the framework of “OLAF” project [41]). This group included 2798 (16.32%) children, among them 9.17% of 6-year olds, 19.30% of 10-year olds and 20.48% of 14-year olds. All these children were enrolled to intervention and education programme. A total of 1627 children who have been qualified on the basis of the screening, eventually took part in the first edition of the programme.

The intervention included four appointments with participating children and their guardians: at enrolment, as well as 3, 6 and 12 months thereafter. The appointments had form of individual consultations with various specialists. The first visit included complex medical examination, discussion on health status of a child, interpretation of laboratory findings, and consultation with a dietician, psychologist and physical education specialist. Then, individual protocol of health intervention was developed for each subject. The aim of the intervention was to implement dietary modifications, to enhance health activity of the child, and to reinforce health-oriented behaviours among his/her family members. Compliance with the protocol and the results of the intervention were verified during follow-up meetings with the specialists, and used as a basis for further tailor-made management.

Interim evaluation of the programme’s effectiveness included a group of 300 children. Mean BMI percentiles at enrolment and following 12 months of the intervention were 92.96 and 88.83, respectively. The tailor-made intervention contributed to a significant decrease in both BMI percentile (*p* = 0.0001) and fat mass content determined by means of bioimpedance (from 39.0% to 31.4%, *p* = 0.0001).

Based on these findings, the municipality of Gdansk decided to support the programme during subsequent years.

#### Intervention

26-week vitamin D supplementation (1200 IU) in obese children presenting with low serum concentrations of 25OHD3 at enrolment to the integrated weight-loss programme.

#### Study protocol

This is a double-blind randomized study. Upon enrolment, the subjects were randomized to one of the two groups.

#### Objectives

To our best knowledge, this is the first randomized placebo-controlled study analysing the effects of vitamin D supplementation in obese children subjected to a weight-loss intervention. The aims of the study were:

1. to analyse the effect of vitamin D supplementation on BMI, body composition and bone mineral density of obese children participating in the weight-loss programme;
2. to analyse the effect of vitamin D supplementation on the risk profile of obesity-related complications, namely impaired glucose tolerance, insulin resistance, dyslipidaemia and arterial hypertension in obese children participating in the weight-loss programme;
3. to analyse the prevalence of vitamin D deficiency in obese children;
4. to verify if selected biological characteristics of obese children (age, sex, pubertal status) may constitute a risk factor for vitamin D deficiency.

#### Primary endpoints

Relative changes in BMI, body composition and bone mineral density in the group of vitamin D-supplemented children, determined after 26 weeks of the intervention.

#### Secondary endpoints

Relative changes in the following parameters in the group of vitamin D-supplemented children and in the placebo group, compared after 26 weeks of the intervention:

1. blood level of vitamin D
2. arterial blood pressure
3. biochemical parameters (lipid profile, oral glucose tolerance test - OGTT, homeostatic model assessment – insulin resistance - HOMA-IR, alanine transaminase - ALT, aspartate transaminase – AST, Calcium - Ca, phosphor - P)
4. Inflammatory markers (high sensivity C reactive protein - hs-CRP, chemerin).

#### Determination of the sample size

The size of the sample (*n* = 100 + 100) was estimated for bi-factorial analysis of variance (2) group x (2) time with main effect and interaction analysis, with target statistical power of 0.90 and significance of differences at *p* < 0.05. Assuming probability of the event (at least 10% reduction in baseline BMI over the follow-up period) at 0.85 and 0.6 for the experimental and control group, respectively, minimum sample size providing 0.9 statistical power for alpha equal 0.05 or lower and beta equal 0.1 or lower was estimated at 130 (65 per group). These values were rounded up to 100 per group assuming up to 30% drop-out rate.

Two hundred participants have been randomly assigned to one of the two groups:

- **GROUP I:** medical intervention, intervention of a dietician, psychologist and specialist of physical education, parental education + oral administration of vitamin D3 (1200 IU per day) for 26 weeks
- **GROUP II:** medical intervention, intervention of a dietician, psychologist and specialist of physical education, parental education + daily oral administration of placebo for 26 weeks.

### Selection method

#### First stage of the selection

Children aged 6, 10 and 14 years, attending primary and grammar schools in Gdansk are subjected to a screening and intervention programme subsidized by the municipality of Gdansk (approved by the Local Ethics Committee, decision no. NKBBN/228/2012). Participants whose anthropometric parameters correspond to overweight (BMI between the 85th and 95th percentile) or obesity (BMI > =95th percentile), according to the percentile charts developed within the framework of the “OLAF” project [41], are qualified to a complex educational and medical intervention for children at increased risk of civilization-related disorders. The aim of the programme is to reduce baseline body weight of participating children by at least 5%.

#### Second stage of the selection

All enrolled children whose parents gave consent for their participation in the “6–10-14 for Health” programme are subjected to laboratory testing (complete blood count, lipid profile, ALT and AST activity, oral glucose tolerance test, creatinine, insulin and thyroid hormone levels) and paediatric consultation. Blood concentration of 25(OH)D3 is determined as well. Finally, an additional 4-ml blood sample is collected and stored for the purpose of further laboratory testing.

#### Third stage of the selection

Upon obtaining a written consent from their parents or guardians, all children with obesity or overweight and decreased blood concentration of 25(OH)D3 are enrolled.

Inclusion criteria

- overweight (BMI between the 85th and 95th percentile) or obesity (BMI > =95th percentile), identified on the basis of anthropometric parameters
- blood concentration of 25(OH)D3 < 30 ng/ml
- written informed consent from legal guardians

Exclusion criteria

- Chronic conditions (asthma or allergies, inflammatory diseases of connective tissue, gastrointestinal disorders, diseases of kidneys and liver, disorders of bone metabolism)
- Contraindications to administration of vitamin D
- Administration of any preparation containing vitamin D, calcium, or steroid hormones during 3 months preceding the study

**Fig. 1:**
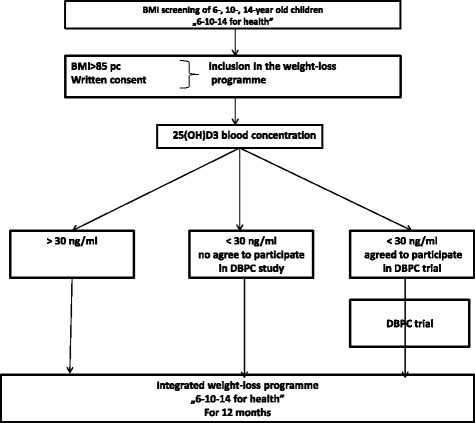
Patient flow chart

**Fig. 2:**
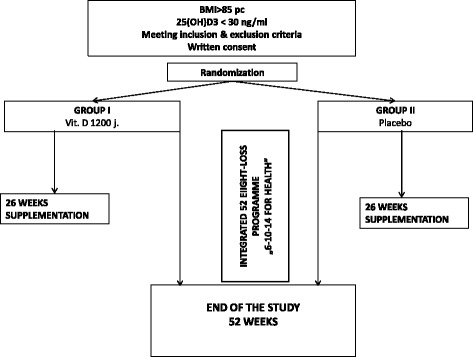
Study flow chart

Computer generated random numbers were given to vitamin D or placebo packages at the time of their appearance at the University Clinical Center Clinical Trial Office (Figs. 1 and 2).

#### Treatment dispensing, assignment of interventions and assessment of compliance

Both of the study treatments: vitamin D (1200 IU) and placebo were provided from the vendor (Sequoia) as identical capsules placed in the same looking packages (5 capsules per blister, 6 blisters per box). The sets of 7 boxes (corresponding to 6-month treatment) are prepared and blinded at the University Clinical Centre Clinical Trial Office by an independent worker, who does not take part in the study. After assigning the numbers packages are handed out to the subjects by the investigator (Medical Doctor) during the enrolment visit, the investigator enrols the patients to the study without knowing the group assignment. The investigator and the patient is blinded for the intervention. During the last visit, the investigator collects all blisters and boxes, to assess treatment compliance on the basis of the remaining capsule number.

### Study methods

**Weight- loss intervention programme**

- Dietetic consultation – analysis of nutritional behaviours and dietary habits, selection of appropriate diet. Dietetic consultation takes place at enrolment and 3, 6 and 12 months thereafter.
- Psychological consultation – reinforcement of changes in health behaviours of the child and his/her parents.
- Consultation with a physical education specialist – defining optimal level of physical activity, adjusted to the subject’s body weight, abilities and preferences, development of a training programme with increasing intensity and volume of physical activity.

### Measurements

#### Anthropometry

Body weight and body height are determined with a digital scale (Mensor WE150, Poland), with the child wearing an underwear and standing barefoot. Body height is measured to the nearest 0.001 m, and body weight to the nearest 0.1 kg. The scale is calibrated every day. Waist and hip circumferences are measured on a horizontal plane by an Ergonomic Circumference Measuring Tape (model 201; Seca GmbH & Co, KG, Hamburg, Germany).

#### Blood pressure measurement

Arterial blood pressure is determined oscillometrically (Omron) on the left arm, with a cuff of an adequate size placed at the level of the heart, in the child seated with uncrossed legs, following at least a 5-min rest in the seated position. The width of the inflatable cuff corresponds to at least 40% of arm circumference. Three separate measurements of blood pressure are taken and averaged [42].

#### Kasch pulse recovery step test

The participants are subjected to a 3-min Kasch pulse recovery (KPR) step test [43, 44]. The test consists of climbing a 0.305 m step at a rate of 24 steps-up/−down per minute. The rate of climbing is defined by a metronome set at 96 beats (signals) per minute. Heart rate (HR) is monitored continuously with “Polar” (Finland) electronic analyser for 3 min of the exercise (step-test) and during 1 min and 5 s of recovery in a seated position. Only post-exercise HR recorded within one minute, starting 5 s after completing the test, is subjected to analysis. All HR characteristics are recorded during restitution in a seated position (subjects are instructed to sit still, breath normally and not involve in a conversation). An arithmetic mean calculated from these values is subjected to further analyses.

#### Dietetic assessment

Dietary records from three consecutive days (2 weekdays +1 day of a weekend), collected prior to enrolment and at the end of the intervention are analysed, and dietary intakes of calcium and vitamin D are calculated with Dieta 5.0 software (Institute of Food and Nutrition, Warsaw).

**Pubertal status** is determined on based on the results of physical examination and expressed using the Tanner stage.

#### Dual-energy X-ray absorptiometry (DXA)

Total body bone mineral content (TBBMC), total body bone mineral density (TBBMD), lean body mass (LBM), fat mass (FT) (Hologic Discovery Wi).

**Laboratory parameters:**

Complete blood count.

Lipid profile determined with an enzymatic method.

Oral glucose tolerance test (OGTT) with glucose concentration determined with hexokinase method.

Concentration of insulin determined by means of an immunochemiluminescence assay.

Concentration of creatinine.

ALT, TSH, fT4, PTH, Ca, P,

hs-CRP level determined by means of an immunoturbidimetric assay.

Concentration of 25(OH)D3 determined by means of an immunochemiluminescence assay.

Visit 1 (enrolment)

- medical history, physical examination
- interpretation of laboratory findings (tests conducted during screening + concentration of vitamin D),
- anthropometric evaluation and analysis of body composition (bioimpedance method)
- consultation with a dietician, psychologist and specialist in physical activity, and defining detailed protocol of the intervention
- Kasch Pulse Recovery Test
- determination of hs-CRP, IL-6, chemerin and adiponectin concentrations in blood samples obtained during screening
- familiarizing child’s parents with the objectives and protocol of the study
- obtaining written informed consent for participation in the study
- randomization to GROUP I or II
- densitometry (DXA) – within one week after enrolment

Visit 2 (3 months)

- medical history, physical examination
- anthropometric evaluation and analysis of body composition (bioimpedance method)
- consultation with a dietician, psychologist and specialist in physical activity, and defining detailed protocol of the intervention. Within a week prior to Visit III – obtaining blood (ca. 5 ml) for laboratory testing (complete blood count, lipid profile, hs-CRP, OGTT, insulin, IL-6, adiponectin and chemerin concentrations).

Visit 3 (6 months)

- medical history, physical examination
- interpretation of laboratory findings
- anthropometric evaluation and analysis of body composition (bioimpedance method)
- consultation with a dietician, psychologist and specialist in physical activity, and defining detailed protocol of the intervention
- obtaining blood for laboratory testing
- termination of active compound/placebo administration

Visit 4 (12 months)

- medical history, physical examination
- anthropometric evaluation and analysis of body composition (bioimpedance method)
- consultation with a dietician, psychologist and specialist in physical activity, and defining detailed protocol of the intervention
- densitometry (DXA) – within one week after the study termination (Table 1)

**Table 1:** Investigation schedule

Procedure	Screening	Inclusion	3 Months	6 Months	12 Months
Antropomethry	X				
Qualification to study	X				
Serum 25(OH)D3	X				
Blood morphology		X			X
Lipid profile		X			X
OGTT (oral glucose tolerance test)		X			X
Insulin during OGTT		X			X
Creatinine		X			X
ALT		X			X
Electrolyte (Na, K)		X			X
TSH		X			X
fT4		X			X
PTH		X			X
Ca		X			X
P		X			X
Hs-CRP		X			X
Chemerin		X			X
Adiponectin		X			X
25(OH)D3					X
DXA		X			X
Pediatric consultation		X	X	X	X
Bioimpedance		X	X	X	X
Anthropometry			X	X	X
Dietetic consultation		X	X	X	X
Calcium and vit. D in diet ary assessment		X	X	X	X
Physical activityconsultation		X	X	X	X
Kash pulse recovery tesr		X	X	X	X
Psychologic consultation		X	X	X	X

Statistical analysis will include:

- verification of normal distribution of continuous variables with the Shapiro-Wilk test.

- determination of basic statistical characteristics for continuous and discrete variables.

- intergroup comparisons with parametric and non-parametric tests.

- intragroup comparisons with parametric and non-parametric tests.

- analysis of intervention efficacy based on bi-factorial analysis of variance (2) group x (2) time with main effect and interaction analysis.

#### Finance

The study is supported by Nutricia Foundation RG-1/2015 (contact person: Zuzanna Janakowska, e-mail: biuro@fundacjanutricia.pl).

## Discussion

The study is currently ongoing and the data are still collected. No partial results have been published thus far. Obesity, defined as an excessive accumulation of adipose tissue with resultant impairment of body functioning, is associated with an increase in morbidity and mortality risk. A dramatic increase in the prevalence of overweight and obesity among children has been documented during recent 30 years. A study conducted at the Institute of Food and Nutrition within the framework of the National Programme for Obesity Prevention showed that about 12–14% of Polish children are obese [45], and in another study performed by Kułaga et al. in 2007–2009, the prevalence of overweight and obesity among boys and girls was estimated at 18.7% and 14.1%, respectively [46, 47]. Also our own data obtained within the framework of the “6–10-14 for Health” programme imply that the problem of excess body weight refers to 15.6% of paediatric population in Gdansk.

Many authors emphasized the importance of a complex approach to children with obesity. Also involvement of family members, friends and acquaintances is considered to be an important determinant of a successful weight-loss intervention [1–3].

Our preliminary findings imply that 12-month intervention is sufficient for a significant decrease in absolute BMI value, BMI percentile and fat mass content (*p* = 0.0001).

The hereby presented study is conducted within the framework of a well-established programme, and we benefit from our previous 5-year experiences with this project.

The study protocol will enable us to estimate the prevalence of vitamin D deficiency in obese children from Gdansk and to compare this figure with available epidemiological data for other populations [7–9]. Aside from a decrease in physical activity level, overt inflammatory response and hormonal dysregulation, excess body weight likely affects also bone metabolism and bone mineral density. However, available data in this matter are still inconclusive [19–26]. Importantly, a link between obesity and bone mass/bone density has not been confirmed in any large population-based study. The discrepancies between the results of previous studies may reflect methodological differences (e.g. measurement of bone density in various segments of the skeleton) or individual variability in bone maturation rate. Further, available evidence regarding the effect of weight loss on bone mineral density is generally sparse, inconclusive and limited mostly to adults [32–35].

Still little is known about the effects of vitamin D supplementation on the outcome of a weight-loss intervention in obese subjects. All published studies dealing with the problem in question included exclusively adults [38, 40] and centred around BMI and fat mass only [38, 39]. One recent study analysed weight loss-related changes in bone mineral density of 50–75-year-old subjects [40]; to the best of our knowledge, this is the only report documenting potential beneficial effects of vitamin D supplementation in prevention of bone mass loss during body weight reduction. The hereby presented project seems to be justified in view of a metabolic diversity typical for the puberty; in our opinion, this study may provide an important insight into biological effects of vitamin D supplementation in obese children being enrolled to an integrated multidisciplinary program for body weight reduction.

It is noteworthy that the results of this study will likely find an application in everyday clinical practice. Providing adequate evidence from this project, monitoring of vitamin D level and its supplementation in deficient subjects may become an imperative in paediatric patients with excess body weight.

Patient flow chart depicts the process of qualifying participants of the “6–10-14 for Health” programme to the study. As all children participating in the programme are overweight/obese, blood concentrations of 25(OH) D3 will be determined, and only the subjects with vitamin D3 levels below 30 ng/ml will be enrolled, providing consent from their parents/legal guardians. Then, all the subjects will be managed in line with the “6–10-14 for Health” programme protocol.

The flow chart depicts the procedure and scheme of qualification, randomization, duration of the study and included interventions.

The table provides detailed information about the procedures (medical examination, laboratory tests) included at consecutive stages of the study.

## Abbreviations

25(OH)D3: 25-hydroxyvitamin D, vitamin D
ALT: Alanine transaminase
AST: Aspartate transaminase
BMI: Body mass index
Ca: Calcium
CRP: C reactive protein
DXA: Dual-energy X-ray absorptiometry
FT: Fat mass
HbA1c: Glycated haemoglobin
HOMA-IR: Homeostatic model assessment – insulin resistance
HR: Heart rate
hs-CRP: High sensitivity C reactive protein
IL-6: Interleukine 6
KPR: Kasch pulse recovery step test
LBM: Lean body mass
OGTT: Oral glucose tolerance test
P: phosphorus
PTH: Parathyroid hormone
TBBMC: Total body bone mineral content
TBBMD: Total body bone mineral density
TNF-α: Tumour necrosis factor α

## References

[1] Nemet D, Levi L, Panatowitz M, Eliakim A (2014). A combined nutritional-behavioral-physical intervention for the treatment of childhood obesity – a 7-year summary. J Pediatr Endocrinol Metab.

[2] Oude Luttikhuis H, Baur L, Jansen H, Shrewsbury VA, O’Malley C, Stolk RP, Summerbell CD (2009). Interventions for treating obesity in children. Cochrane Database Syst Rev.

[3] Masquio DC, de Piano A, Campos RM, Sanches L, Carnier J, Corgosinho FC (2015). The role of multicomponent therapy in the metabolic syndrome, inflammation and cardiovascular risk in obese adolescents. Br J Nutr.

[4] De Lima SP, de Mello MT, Elias N, Fonseca FA, de Piano A (2011). Improvement in HOMA-IR is an independent predictor of reduced carotid intima-media thickness in obese adolescents participating in an interdisciplinary weight-loss program. Hypertens Res.

[5] Masquio DC, de Piano A, Sanches PL, Corgosinho FC, Campos RM, Carnier J (2013). The effect of weight loss magnitude on pro−/anti-inflammatory adipokines and carotid intima-media thickness in obese adolescents engaged in interdisciplinary weight loss therapy. Clin Endorinol (Oxf).

[6] Reinehr T (2013). Calculating cardiac risk in obese adolescents before and after onset of life style intervention. Expert Rev Cardiovasc Ther.

[7] Aypak C, Turedi O, Yuce A (2014). The association of vitamin D status with cardiometabolic risk factors, obesity and puberty in children. Eur J Pediatr.

[8] Lee SH, Kim SM, Park HS, Choi KM, Cho GJ, Ko BJ (2013). Serum 25-hydroxyvitamin D levels obesity and the metabolic syndrome among Korean children. Nutr Metab Cardiovasc Dis.

[9] Sioen I, Mouratidou T, Kaufman JM, Bammann K, Michels N, Piget I (2012). IDEFICS consortium. Determinants of vitamin D status in young childre: results from the Belgian arm of the IDEFICS study. Public Health Nutr.

[10] Kamycheva E, Joakimsen RM, Jorde R (2003). Intakes of calcium and vitamin d predict body mass index in the population of northern Norway. J Nutr.

[11] Kull M, Kallikorm R, Lember M (2009). Body mass index determines sunbathing habits: implications on vitamin D levels. Intern Med J.

[12] Pacifico L, Anania C, Osborn JF, Ferraro F, Bonci E, Olivero E (2011). Low 25(OH)D3 levels are associated with total adiposity, metabolic syndrome, and hypertension in Caucasian children and adolescents. Eur J Endorinol.

[13] Rosenstreich SJ, Rich C, Volwiler W (1971). Deposition in and release of vitamin D3 from body fat: evidence for a storage site in the rat. J Clin Invest.

[14] Verrijn Stuart AA, van Summeren M, Rakhshandehroo M, Nuboer R, de Boer FK (2014). Vitamin D deficiency in childhood obesity is associated with high levels of circulating inflammatory mediators, and low insulin sensitivity. Int J Obes.

[15] Ganji V, Zhang X, Shaikh N, Tangpricha V (2011). Serum 25-hydroxyvitamin D concentrations are associated with prevalence of metabolic syndrome and various cardiometabolic risk factors in US children and adolescents based on assay-adjusted serum 25-hydroxyvitamin D data from NHANES 2001-2006. Am J Clin Nutr.

[16] Song Y, Wang L, Pittas AG, Del Gobbo LC, Zhang C, Manson JE (2013). Blood 25-hydroxy vitamin D levels and incident type 2 diabetes: a meta-analysis of prospective studies. Diabetes Care.

[17] Almezadeh R, Kichler J, Babar G, Calhoun M (2008). Hypovitaminosis in obese children and adolescents: relationship with adiposity, insulin sensitivity, ethnicity, and season. Metabolism.

[18] Belenchia AM, Tosh AK, Hillman LS, Peterson CA (2013). Correcting vitamin D insufficiency improves insulin sensitivity in obese adolescents: a randomized controlled trial. M J Clin Nutr.

[19] Goulding A, Taylor RW, Jones IE, McAuley KA, Maning PJ, WIliams SM (2000). Overweight and obese chilldren have low bone mass and area for their weight. Int J Obes Relat Metab Disord.

[20] El Hage R, Jacob C, Moussa E, Benhamou CL, Jaffre C (2009). Total body, lumbar spine and hip bone mineral density in overweight adolescent girls: decreased or increased?. J Bone Miner Metab.

[21] El Hage R, El Hage Z, Jacob C, Moussa E, Theunynck D, Baddoura R (2011). Bone mineral content and density in overweight and control adolescent boys. J Clin Desitom.

[22] Leonrd MB, Shults J, Wilson BA, Terhakovec AM, Zemel BS (2004). Obesity during childhood and adolescence augments bone mass and bone dimension. Am J Clin Nutr.

[23] Kessler J, Koebnick C, Smith N, Adams A (2013). Childhood obesity is associated with increased risk of most lower extremity fractures. Clin Orthop Relat Res.

[24] Liu K, Liu P, Liu R, Wu X, Cai M (2015). Relationship between serum leptin levels and bone mineral density: a systematic review and meta-analysis. Clin Chim Acta.

[25] Maggio AB, Belli DC, Puigdefabregas JW, Farpour-Lambert NJ, Beghetti M, McLin VA (2014). High bone density in obese adolescents is related to fat mass and serum leptin concentration. J Pediatr Gastroenterol Nutr.

[26] Rosen CJ, Bouxsein ML (2006). Mechanisms of disease: is osteoporosis the obesity of bone?. Nat Clin Pract Rheumatol.

[27] Cashman KD, Hill TR, Cotter AA, Boreham CA, Dubitzky W, Murray L, Strain J (2008). Low vitamin D status adversly affects bone health parameters in adolscents. Am J Clin Nutr.

[28] Pekkinen M, Vilijakinen H, Saarnio E, Lamberg-Allardt C, Makitie O (2012). Vitamin D is a major determinant of bone mineral density at school age. PLoS One.

[29] Wizenberg T, Powell S, Shaw KA, Jones G (2011). Effects of vitamin D supplementation on bone density in healthy children: systemie review and meta-analysis. BMJ.

[30] El-Hajj Fuleihan G, Nabulsi M, Tamim H, Maalouf J, Salamoun M, Khalife H (2006). Effect of vitamin D repalcement on musucoskeletal parameters in school children: a randomized controlled trial. J Clin Endocrinol Metab.

[31] Lenders CM, Feldman HA, Von Scheven E, Merewood A, Sweeney C, Wilson DM (2009). Relation of body FAT indexes to vitamin D status and defeciency among obese adolescents. Am J Clin Nutr.

[32] Soltani S, Hunter GR, Kazemi A, Shab-Bidar S. The effects of weight loss approaches on bone mineral density in adults: a systematic review and meta-analysis of randomized controlled trials. Osteoporos Int 2016;6. [Epub ahead of print] 10.1007/s00198-016-3617-4 27154437

[33] Rector RS, Loethen J, Ruebel M, Thomas TR, Hinton PS (2009). Serum markers of bone turnover are increased by modest weight loss with or without weight-bearing exercise in overweight premenopausal women. Appl Physiol Nutr Metab.

[34] Shapses SA, Von Thun NL, Heymsfield SB, Ricci TA, Ospina M, Pierson RN, Stahl T (2001). Bone turnover and density in obese premenopausal women during moderate weight loss and calcium supplementation. J Bone Miner Res.

[35] Yu EW, Bouxsein ML, Putman MS, Monis EL, Roy AE, Pratt JS (2015). Two-year changes in bone density after roux-en-Y gastric bypass surgery. J Clin Endocrinol Metab.

[36] Kaulfers AM, Bean JA, Inge TH, Dolan LM, Kalkwarf HJ (2011). Bone loss in adolescents after bariatric burgery. Pediatrics.

[37] Labouesse MA, Gertz ER, Piccolo BD, Souza EC, Schuster GU, Witbracht MG (2014). Associations among endocrine, inflammatory and bone markers, body composition and physical activity to weight loss induced bone loss. Bone.

[38] Salehpour A, Hosseinpanah F, Shidfar F, Vafa M, Razaghi M, Dehghani S (2012). A 12-week double-blind randomized clinical trial of vitamin D3 supplementation on body fat mass in healthy overweight and obese women. Nutr J.

[39] Mason C, Xiao L, Imayama I, Duggan C, Wang CY, Korde L, et al. Vitamin D3 supplementation during weight loss: a double-blind randomized controlled trial. Am J Clin Nutr. 2014. (pub ahead of print). 10.3945/ajcn.113.073734 PMC3985208 24622804

[40] Mason C, Tapsoba JD, Duggan C, Imayama I, Wang CY, Korde L (2016). Effects of vitamin D3 supplementation on lean mass, muscle strength, and bone mineral density DuringWeight loss: a double-blind randomized controlled trial. J Am Geriatr Soc.

[41] Kułaga Z, Różdżyńska A, Palczewska I, Grajda A, Gurzkowska B, Napieralska E (2010). Siatki centylowe wysokości, masy ciała i wskaźnika masy ciała dzieci i młodzieży w Polsce – wyniki badania OLAF. Standardy Medyczne.

[42] O’ Brien E, Parati G, Stergiou G, Asmar R, Beilin L, Bilo G (2013). European society of hyprtension position paper on ambulatory blood pressure monitoring. J Hypertens.

[43] Cole CR, Blackstone EH, Pashkow FJ, Snader CE, Lauer MS (1999). Heart-rate recovery immediately after exercise as a predictor of mortality. N Engl J Med.

[44] Jankowski M, Niedzielska A, Brzezinski M, Drabik J (2015). Cardiorespiratory fitness in children: a simple screening test for population studies. Pediatr Cardiol.

[45] Narodowy Program Zapobiegania Nadwadze i Otyłości oraz Przewlekłym Chorobom Niezakaźnym Poprzez Poprawę Żywienia i Aktywności Fizycznej na lata 2007-2011. Ministerstwo Zdrowia, Departament Polityki Zdrowotnej.

[46] Kułaga Z, Litwin M, Tkaczyk M, Palczewska I, Zajączkowska M, Zwolińska D (2011). Polish 2010 growth references for school-aged children and adolescents. Eur J Pediatr.

[47] Kułaga Z, Litwin M, Zajączkowska M, Wasilewska A, Morawiec-Knysak A, Różdżyńska A (2008). Porównanie wartości obwodów talii i bioder dzieci i młodzieży polskiej w wieku 7-18 lat z wartościami referencyjnymi dla oceny ryzyka sercowo-naczyniowego - wyniki wstępne projektu badawczego OLAF (PL0080). Standardy Medyczne Pediatria.

